# *In Vitro* Antioxidant, Anticoagulant and Antimicrobial Activity and in Inhibition of Cancer Cell Proliferation by Xylan Extracted from Corn Cobs

**DOI:** 10.3390/ijms13010409

**Published:** 2011-12-29

**Authors:** Raniere Fagundes Melo-Silveira, Gabriel Pereira Fidelis, Mariana Santana Santos Pereira Costa, Cinthia Beatrice Silva Telles, Nednaldo Dantas-Santos, Susana de Oliveira Elias, Vanessa Bley Ribeiro, Afonso Luis Barth, Alexandre José Macedo, Edda Lisboa Leite, Hugo Alexandre Oliveira Rocha

**Affiliations:** 1 Laboratory of Biotechnology of Natural Polymers (BIOPOL), Department of Biochemistry, Federal University of Rio Grande do Norte (UFRN), Avenue Salgado Filho S/N, Natal-RN 59078-970, Brazil; E-Mails: ranierefagundes@hotmail.com (R.F.M.-S.); gabrielfidelis_@hotmail.com (G.P.F.); marispc_bio@yahoo.com.br (M.S.S.P.C.); cinthiatelles@yahoo.com.br (C.B.S.T.); nednaldod@hotmail.com (N.D.-S.); 2 Biotechnology Center and Faculty of Pharmacy, Federal University of Rio Grande do Sul (UFRGS), Rio Grande do Sul, Porto Alegre, 90690-000, Brazil; E-Mails: susanaelias@gmail.com (S.O.E.); alexandre.macedo@ufrgs.br (A.J.M.); 3 Faculty of Pharmacy and Porto Alegre Clinical Hospital, Federal University of Rio Grande do Sul (UFRGS), Rio Grande do Sul, Porto Alegre, 90690-000, Brazil; E-Mails: vanebley@hotmail.com (V.B.R.); albarth@hcpa.ufrgs.br (A.L.B.); 4 Laboratory of Glycobiology, Department of Biochemistry, Federal University of Rio Grande do Norte (UFRN), Avenue Salgado Filho S/N, Natal-RN 59078-970, Brazil; E-Mail: eddaleite@cb.ufrn.br

**Keywords:** corn cob food, antimicrobial, heteroxylan, antioxidant polysaccharide, HeLa cells, 3T3 cells

## Abstract

Xylan is one of most abundant polymer after cellulose. However, its potential has yet to be completely recognized. Corn cobs contain a considerable reservoir of xylan. The aim of this work was to study some of the biological activities of xylan obtained from corn cobs after alkaline extraction enhanced by ultrasonication. Physical chemistry and infrared analyses showed 130 kDa heteroxylan containing mainly xylose:arabinose: galactose:glucose (5.0:1.5:2.0:1.2). Xylan obtained exhibited total antioxidant activity corresponding to 48.5 mg of ascorbic acid equivalent/g of xylan. Furthermore, xylan displayed high ferric chelating activity (70%) at 2 mg/mL. Xylan also showed anticoagulant activity in aPTT test. In antimicrobial assay, the polysaccharide significantly inhibited bacterial growth of *Klebsiella pneumoniae*. In a test with normal and tumor human cells, after 72 h, only HeLa tumor cell proliferation was inhibited (*p* < 0.05) in a dose-dependent manner by xylan, reaching saturation at around 2 mg/mL, whereas 3T3 normal cell proliferation was not affected. The results suggest that it has potential clinical applications as antioxidant, anticoagulant, antimicrobial and antiproliferative compounds.

## 1. Introduction

Reactive oxygen species (ROS) are essential for life because they are involved in cell signaling and used also by phagocytes for their bactericidal action. However, nonessential production of ROS, such as oxidative stress, is suggested to be strongly associated with the aging process and certain degenerative diseases, including various cancers, cognitive dysfunctions, and coronary heart disease [1]. Antioxidative materials have recently attracted considerable attention because of their potential as reactive oxygen species scavengers [2]. Thus, many antioxidants have been identified for use as food additives or medical supplements as reactive oxygen species scavengers that will nullify the harmful effects from ROS.

Synthetic antioxidants such as butylated hydroxytoluene (BHT), butylated hydroxyanisole (BHA), and propyl gallate have been used as food antioxidants. However, the use of these synthetic antioxidants is negatively perceived by consumers due to their potential toxicity and connotation as chemicals in food [3]. Natural antioxidants have awakened a great deal of interest because of their health effects and positive image as substances acting against degenerative diseases and certain cancers. Therefore, owing to consumer concerns about the safety of synthetic antioxidants, the promotion of natural antioxidants has been greater than ever [4]. Plants are the main source of natural antioxidants, including several edible species. Among antioxidant molecules, tocopherols, carotenoids, ascorbic acids, and phenolic compounds stand out. Additionally, the antioxidants present in fruits and vegetables have been found to exhibit anticarcinogenic and antimutagenic [5].

Recently, several carbohydrates, including sulfated polysaccharides from seaweeds [6], alginate, and chitosan have been reported to possess antioxidant and antiproliferative properties [7]. Carbohydrates with antiproliferative and antioxidant effects are likely to be very useful, as they generally exhibit high water solubility and low toxicity. Moreover, most of these molecules have been safely consumed as components of food products for many years. Considerable attention has recently been paid to the development of novel carbohydrate additives with potent antiproliferative and/or antioxidative activity [8].

Plant extracts have been used for thousands of years for a variety of human diseases, including infection by bacteria [9]. With the increasing of cases of bacteria resistant to a wide number of antibiotic agents, there is great concern in the search for new compounds with antibacterial activity [10]. However, little is known about the antimicrobial properties of polysaccharides, including xylans from corn cobs, so this work also aims to expand the limited knowledge about it.

The β(1–4)-d-xylan-type hemicelluloses are the second most abundant biopolymer, after cellulose, in annual plants and hardwoods. They represent an important renewable biopolymer resource whose potential has yet to be completely realized. Xylans are somewhat complex polysaccharides whose structures vary with the plant and plant tissue source [11]. Corn cobs contain a considerable reservoir of xylan-type hemicelluloses. The estimate for world maize production in 2010/2011 was approximately 810 million tons. The global demand to the same period is approximately 840 million tons and estimates of corn cob production, a subproduct of maize production, is approximately the same amount. The market value of maize worldwide is more than US$ 150 billion/year. Therefore, there is a great incentive to further exploit corn and its subproducts in order to provide a more complete characterization of its benefits. In addition, the fact that corncobs are currently being used as ground flour to produce bread and as a stabilizer for edible oil increases its acceptance in world markets [12].

Reports have been published concerning elucidation of the structural details of d-xylans from corn cobs [13,14]. Nevertheless, few papers are dedicated to systematically studying the biological properties of these polymers [11,15]. Thus, the aim of this work is to investigate some of the biological activities of water-soluble xylan obtained from corn cobs after alkaline extraction was enhanced by ultrasonication. To that end, *in vitro* xylan antioxidant, anticoagulant (aPTT test), antimicrobial (Antibiofilm and Bacterial growth assay) and antiproliferative activities against HeLa cancer cell lines and normal 3T3 fibroblast cells were evaluated.

## 2. Results and Discussion

### 2.1. Chemical Analysis

Xylan, the most common hemicellulose, accounts for more than 60% of polysaccharides present in the cell walls of corn cobs [16]. Using a methodology that combined alkaline extraction and ultrasound, we obtained water soluble xylan from the corn cobs, yielding 40 ± 5% (w/w). When only alkali solution is used as extractant, xylan extraction yield is about 15% [17]. The beneficial sonication effect on xylan extractability can be explained by both the mechanical disruption of the cell walls and breaking of inter- and intramolecular xylan linkages, enhanced in the presence of hot alkali [18]. As a result, accessibility, solubility and diffusion of dissolved cell wall molecules increased.

We used a method for xylan extraction from corn cobs proposed by Wang and Zhang [14]. However, these authors did not analyze the molecular weight of xylan in their article. The xylan solution obtained in this work was submitted to gel permeation chromatography, showing a peak of 130 ± 20 kDa heteroxylan. This material was pooled and lyophilized and its chemical (Table 1) and biological features analyzed. Three additional peaks composed of small oligosaccharides were also found.

Chemical analysis of xylan is summarized in Table 1. Phenolic and protein content for the corn cob sample was very low, whereas sugar content was elevated, indicating the efficiency of the extraction method. With respect to total sugar, phenolic compounds and protein contents, the sum of the three components found in the xylan does not approach 100%. This is due to the fact that this polymer is highly hygroscopic, absorbing water from the atmosphere very quickly after lyophilization. Furthermore, because of the negative charges of glucuronic acids, metals are not eliminated from xylan structures, even after dialysis. Another important point is the conformation exhibited by this polymer in aqueous solutions, which may capture cations within their structures.

The xylan obtained here is composed mainly of xylose, arabinose, galactose and glucose and trace of mannose and glucuronic acid. These monosaccharides were also described in other studies on xylan extracted from corn cobs [15,17,19].

**Figure 1:**
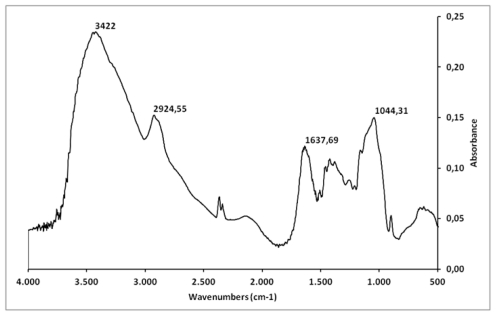
Infrared spectrum of the region between 4000–500 cm^−1^ of polysaccharides from corn cobs. The peaks highlighted with the numbers above show typical signs of heteroxylan and chemical groups such as glucuronic acid, with a peak of 1637 cm^−1^.

The FT-IR spectrum of corn cob xylan is shown in Figure 1. Typical signals of heteroxylan at 3422, 2924, 1637, 1125, 1044, and 895 cm^−1^ were clearly observed in the sample. Xylan exhibited a broad stretching intense characteristic peak at around 3444 cm^−1^ for the hydroxyl group, and a weak band at 2929 cm^−1^ due to CH_2_ and CH_3_ groups [20]. A large peak around 1637 cm^−1^, which overlaps the residual water at 1645 cm^−1^, was assigned to the carboxyl group of glucuronic acid [21,22]. Xylan has a band in the 1200–1000 cm^−1^ region, which is dominated by ring vibrations overlapped with stretching vibrations of C–OH side groups and C–O–C glycosidic band vibration [22]. A specific signal at 1044 cm^−1^ was assigned to the stretching vibration of COH. This signal dominates the xylan spectrum with (1–4)-backbone, moreover, the anomeric region of (1–4)-xylan was assigned at 895 cm^−1^ in accordance with the IR results in xylobioside models [21].

### 2.2. Antioxidant Activity

Total antioxidant activity (TAC) was determined using phosphomolybdenum blue complex. This method is based on the reduction of Mo^+6^ to Mo^+5^ by antioxidant compounds and the formation of green Mo^+5^ complexes with a maximum absorption at 695 nm. The green complex is quite stable for several days and is not affected by various organic solvents used for polysaccharide extraction [23]. Corn cob xylan showed a TAC corresponding to 48.5 mg of ascorbic acid equivalent/g of xylan. This activity was similar to that observed for sulfated polysaccharides from the edible red seaweed *Gracilaria caudata* [8] and higher than the activity of tamarind seed polysaccharide [24]. Due to the higher TAC, corn cob xylan was selected for further detailed analysis of its antioxidative properties, using superoxide radical scavenging activity assay, hydroxyl radical scavenging activity assay, iron-chelating capacity, and reducing power.

Superoxide anion radicals act as an oxidant but are highly unstable, immediately dismutating in the intracellular environment either spontaneously or enzymatically. Although superoxide is a relatively weak oxidant, it decomposes to form stronger reactive oxidative hydroxyl radical species. Among reactive oxygen species (ROS), the hydroxyl radical is the most reactive in chemistry. It can abstract hydrogen atoms from biological thiol molecules and form sulfur radicals capable of combining with oxygen to generate oxysulfur radicals and damage biological molecules [25]. Corn cob xylan (from 0.05 to 2.00 mg/mL) did not show scavenging activity against these ROS. Similar data were observed with commercial xyloglucans, which showed no superoxide or hydroxyl radical scavenging activity (from 0.1 to 1.6 mg/mL). However, when two types of xyloglucan derivatives (xyloglucan selenious ester and sulfated xyloglucan) were prepared from that commercial xyloglucan, they showed stronger superoxide and hydroxyl radical scavenging activities [26]. In fact, fucoidans (sulfated polysaccharides) from the seaweed *Laminaria japonica* [27] and ulvans (sulfated polysaccharides) from the seaweed *Ulva pertusa* [28] exhibited much stronger scavenging activity in the superoxide radical than vitamin C. In addition, the last two studies proposed that scavenging ability depends on sulfate content. Moreover, when chitosan was sulfated, it exhibited high scavenging activity as compared to the original compound [29]. The xylan corn cob also did not show any activity in the reducing power tests at all concentrations used (from 0.05 to 2.00 mg/mL). The presence of sulfate in the polysaccharide structure is also related to reducing power. Several studies show high reducing power activity of sulfated polysaccharides extracted from different sources [27,28]. In fact, the presence of the sulfated group decreases the bond energy of the C–H in the vicinity of the glycosidic bond, and then increases the hydrogen atom donating capability. Therefore, the introduction of sulfate group might enhance the electron cloud density of active hydroxyl groups and enhance the molecular electron-withdrawing activity, which can eliminate free radicals and terminate radical-mediated oxidative chain reactions [30]. However, several authors showed that scavenging activity and reducing power of sulfated polysaccharides is depend on the degree and position of sulfation [8,29,30]. Therefore, further to this study the xylan from corn cob have been selected for further sulfation, including sulfation at different positions of the monosaccharide chain, in order to improve its scavenging activity and reducing power.

**Figure 2:**
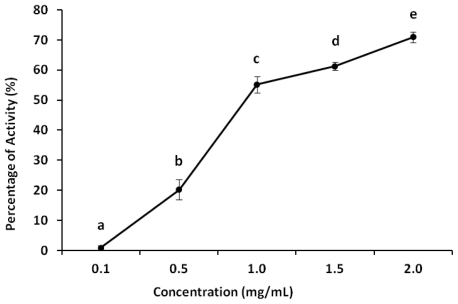
Chelating effect of xylan on ferrous ions. Data are expressed as means ± standard deviation. ^a,b,c,d,e^ Different letters indicate a significant difference between xylan concentrations by one-way Anova followed by Student-Newman-Keuls test (*p* < 0.05).

Antioxidants inhibit interaction between metals and lipids through the formation of insoluble metal complexes with ferrous ion or the generation of steric hindrance. The iron-chelating capacity test measures the ability of antioxidants to compete with ferrozine in chelating ferrous ion. Activity is measured as absorbance of the red Fe^2+^/ferrozine complex decreases. The plot of iron-chelating capacity as a function of sample concentration is shown in Figure 2. Corn cob xylan showed dose-dependent activity, the highest occurring at 2.0 mg/mL (70%). Xylan activity was actually only 1.47 times lower than EDTA activity at the same concentration under the same experimental conditions (Data not shown).

Ferrous ions are considered to be the most effective pro-oxidants present in food systems. The high chelating effect of this polysaccharide would also be beneficial if it were formulated into foods. The metal-chelating property of this polymer displayed that it might be applied in adsorption, metal ions separation or waste water treatment. Some polysaccharides extracted from seaweed showed lower activity compared with those extracted from corn cobs. The most active compounds were from green seaweeds *Caulerpa prolifera* and *Caulerpa sertularioides* with 69.9 and 57.8% of ferrous chelating, respectively, at 2 mg/mL. In fact, the activity of sulfated polysaccharides from *C. prolifera* was only 1.36 times lower than EDTA activity at the same concentration under the same experimental conditions [8]. Several articles have shown that polysaccharide iron chelating activity depends mainly on the presence of groups such as sulfate, alcohol hydroxyl or carboxyl groups. Even when there is a small amount of uronic acid in polysaccharide composition it may have chelating activity, such as *Zizyphus jujuba* polysaccharide, which, despite containing 5.7% of uronic acid, showed a chelating effect about 75% at a concentration of 2000 μg/mL [31]. However, sulfated polysaccharides showed a weak chelating effect even in high concentration (2000 μg/mL) [8]. In addition, when sulfate groups were added to neutral monogalactoglucan [32] or neutral glucan [33] they did not improve the polysaccharide chelating activity, which indicates that polysaccharide chelating activity is stereo-specific, is depending on spatial patterns of negative groups, and it is not merely a consequence of polysaccharide charge density. It was clear that xylan iron-chelating capacity is dependent on its structural features. Additional investigation will help our understanding of the complete xylan structure, including sequence of monosaccharides, configuration and position of glycosidic linkages, the position of branching points and the structure–function relationship.

### 2.3. Antiproliferative Activity

**Figure 3:**
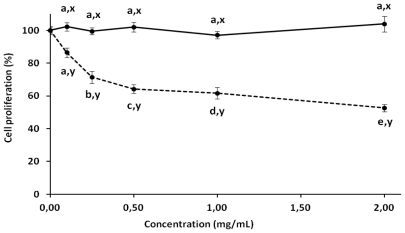
Antiproliferative activity of xylan from corn cobs. Continuous line represents a study of the normal fibroblast line (3T3); dashed line represents a test tumor cell line (HeLa). ^a,b,c,d,e^ Different letters indicate significant differences between different concentrations used in the same cell line; ^x,y^ Different letters represent significant difference between similar concentrations used in different lineages. Student-Newman-Keuls test (*p* < 0.05).

Antiproliferative studies of antioxidant compounds have been reported in recent years. The development of compounds that inhibit or delay tumor cell proliferation and do not affect normal cells is one of the main challenges in the search for antitumor compounds. In this work, corn cob xylan was effective against HeLa tumor cells in a dose-dependent manner, with maximum antiproliferative activity of 50% for about 2 mg/mL. In addition, no activity was found against normal fibroblast cells (3T3) (Figure 3). To the best of our knowledge, there are no data about the antiproliferative activity of xylan from corn cobs. However, polysaccharides from other sources inhibit tumor cell proliferation. Ryu *et al.* reported that 1 mg/mL of purified polysaccharides from *Salicornia herbacea* can inhibit 50% of human colon cancer cell proliferation [34]. The antiproliferative activity of polysaccharides may be attributed to the presence of charged groups attached to the molecule. This was reported by Costa and colleges [8] who showed a relationship between the number of sulfate groups and the antiproliferative action of polysaccharides extracted from seaweed. Moreover, commercial derivatives of xyloglucans (esters of xyloglucans selenium) showed an antiproliferative effect against tumor cells (HepG2) with a 30% inhibition of proliferation with a concentration of 1 mg/mL [26]. However, neutral polysaccharides, containing mainly xylose extracted from Japanese bamboo, manifest selective cytotoxicity against acute lymphoblastic leukemia cells with 60% antiproliferative activity of around 200 μg/mL [35]. This indicates that the antiproliferative activity of polysaccharides, including xylan, is not merely a consequence of their charge density but a set of structural factors of the polysaccharide. No relationship was found between antiproliferative activity and antioxidant content (*R*^2^ = 0.398, *p* > 0.05). Similar results were obtained when antioxidant and anticoagulant activities of aqueous extracts from strawberry were analyzed [36].

### 2.4. Anticoagulant Activity

Assessment of anticoagulant activity is always considered when working with polysaccharides. On the other hand, anticoagulant activity of plant polysaccharides is commonly not evaluated, because they are mostly neutral. No study to date has assessed the anticoagulant activity potential of xylans from corn cobs. It was therefore decided to evaluate the anticoagulant activity of this compound extracted from corn cob flour. To that end, xylan was submitted to anticoagulant tests such as those described in the Experimental Section. Commercial aPTT and PT kits were used to determine if the anticoagulant action of xylan could influence intrinsic and extrinsic coagulation pathways, respectively.

Clotting time of the extrinsic pathway (PT test) showed no increase at any of the conditions used (100, 200, 400, 600 μg). However, a dose-dependent prolongation of clotting time was observed when the intrinsic coagulation pathway was analyzed (aPTT test). It should be noted that with 100 μg of xylan plasma clotting time doubled in relation to the control (Table 2).

**Figure 4:**
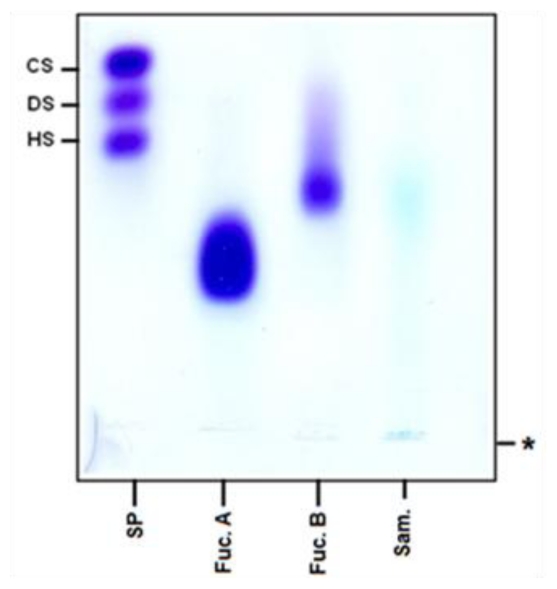
Electrophoretic pattern of xylan from corn cobs. Aliquots of 50 μg of fucan A and B and 5 μg of sulfated glycosaminoglycans, and 100 μg of xylan were applied in agarose gel, PDA buffer 0.05 M, pH 9.0. After precipitation with CETAVLON slides were stained with toluidine blue. SP: Sulfated polysaccharides; CS: chondroitin sulfate; DS: dermatan sulfate; HS: Heparan sulfate; Fuc. A: Fucan A from brown seaweed *Spatoglossum schroederi*; Fuc. B: Fucan B from *Spatoglossum schroederi*; Sam: Sample (xylan); * origin.

Anticoagulant polysaccharides exhibit this activity due to the presence of charges, primarily sulfate groups. Therefore, sulfate concentration was measured, as described in Methods. The sample contained 3.4% sulfate (w/w), which albeit low, might be sufficient for it to exhibit anticoagulant activity. However, the sulfate identified in the sample may not be covalently linked to xylan, but rather to the three dimensional polymer structure. This hypothesis corroborates the fact that the same infrared peaks found in the 800–900 cm^−1^ region of sulfated polysaccharides were not observed. To confirm the link of sulfated groups to the xylan molecule, the latter was submitted to agarose gel electrophoresis. Figure 4 is the digitization of a gel electrophoresis slide after toluidine blue staining. This compound complex with the sulfate groups, forming a purple dye complex, as can be observed with the sulfate polysaccharides used (heparin sulfate, chondroitin sulfate, dermatan sulfate, and sulfate fucans A and B from the brown seaweed *Spatoglossum schroederi*). On the other hand, xylan did not stain purple with toluidine blue, suggesting the absence of sulfate in this molecule.

**Figure 5:**
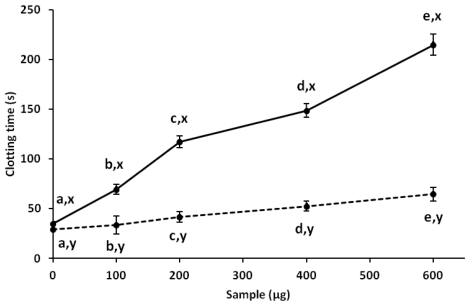
Clotting time using the aPTT test affected by corn cob xylan after carboxy-reduction reaction. Continuous line represents the original xylan and the dashed line represents the xylan after decarboxylation. ^a,b,c,d,e^ Different letters indicate significant differences between different concentrations used; ^x,y^ Different letters represent significant difference between similar concentrations in the different samples used. 1 μg of heparin > 240 s. Student-Newman-Keuls test (*p* < 0.05).

Another charged group may be involved in the anticoagulant activity of xylan, such as carboxyl groups of glucuronic acid residues present in its structure. Yoon and colleges showed that 23 glucuronic acid-containing polysaccharides extracted from 23 different vascular plants have anticoagulant activity. These authors promote the reduction of the glucuronic acid carboxyl groups of the most potent anticoagulant polysaccharide and this abolished its activity. These data showed that glucuronic acid residues are essential for activity since after reduction of its carboxyl groups the anticoagulant activity disappears [37]. Therefore, xylan was submitted to a carboxy-reduction process to evaluate the involvement of carboxyl groups in anticoagulant activity. Analyses indicated a 70% loss of the carboxyls present in the xylan. As a result, this new compound was also submitted to aPTT and PT tests. Evaluation of anticoagulant activity of xylan after carboxy-reduction showed a drastic reduction (Figure 5), indicating the importance of these groups in this activity. These results are in agreement with those obtained by the authors cited above.

### 2.5. Antimicrobial Activity

The microbial resistance to antibiotics is a wide problem of medical importance. Each year, many microbial isolates have been reported as resistant to antibiotics usually used in clinical therapy [10]. This worrisome problem costs annually millions of dollars and is a threat for the human life. Thus, global efforts to understand the biology and biochemistry, and search for new compounds with antibiotic action have been forward in university and research centers around the world. Plant extracts have been used for thousand years to cure a diversity of human illness, including bacterial infection [38]. Therefore, new antibiotic compounds have been discovery and developed from the plant extracts.

In this study, the xylan from corn cob did not inhibit the growth of *Staphylococcus epidermidis* (ATCC 35984). However, the polysaccharide was able to inhibit the growth of one clinical isolate of KPC producing *Klebsiella pneumoniae* in approximately 25%, and consequently the biofilm formation was decreased in a similar proportion (Table 3). KPC isolates are resistant to most antibiotics used to treat clinical infections, in particular, carbapenems. This apparent low activity observed against KPC might be further increased by fractionation or chemical modification of the active compounds, serving the xylan as scaffold molecule. Gul *et al.* have shown that different plant extracts present distinct antimicrobial actions against various different species of bacteria [39]. The difference in this result could possibly be due to the different compilation of receptors in the each species of bacteria that recognize distinct patterns of polysaccharides and thus the way of answers and signaling is unique.

## 3. Experimental Section

### 3.1. Materials

Potassium ferricyanide, ferrous sulfate (II), *n*-propanol, acetic acid, ethanol, and sulfuric acid were obtained from Merck (Darmstadt, Germany). Heparn sulfate, condroitin sulfate, dermatan sulfate, bovine serum albumin and (BSA) sodium chloride were purchased from Sigma Chemical Co. (St. Louis, MO, USA). HeLa cells (ATCC CCL-2) and 3T3 cells (ATCC CCL-92) were a gift from Silvia R. B. Medeiros, Department of Genetic and Cell Biology, UFRN, Brazil. Cell culture medium components [Dulbecoo’s-Minimum essential medium (DMEM)], l-glutamine, sodium bicarbonate, non-essential amino acids, sodium pyruvate, fetal bovine serum (FBS), and phosphate buffered saline (PBS) were purchased from Invitrogen Corporation (Burlington, ON, USA). All other solvents and chemicals were of analytical grade. Fucans A and B from *Spatoglossum schroederi* were obtained as previously described [40,41].

### 3.2. Extraction of Xylan from Corn Cobs

Fresh corn samples, purchased at a market, were cleaned, washed, and completely stripped of grains to avoid contamination. They were further chopped into small pieces, dried, and ground into flour. A volume of 160 mL of methanol was added to 10 g of sample and stirred for 24 h in the dark to eliminate lipids and pigments. The resulting precipitate was collected by centrifugation (10,000 × g, 20 min, 4 °C), vacuum dried, and stored out of the light. Xylan extraction was conducted under the following conditions: corn cob flour was added to NaOH (1.8 M) solution at a ratio of 1:25 (w/v), and the solution was then sonicated at 200 W ultrasound power for 30 min at 5 min intervals and temperature of 60 °C, as described earlier [14]. The solution was centrifuged to separate the extracted material from the insoluble residue (discarded). Four volumes of ice-cold methanol were added to the solution under gentle agitation and maintained at 4 °C for 24 h out of the light. The precipitate formed was collected by centrifugation (10,000 × g, 20 min), vacuum dried, and kept out of the light.

### 3.2. Chemical Analysis and Monosaccharide Composition

Total sugars were estimated by the phenol-H_2_SO_4_ reaction using d-xylose as a standard [8,20]. Protein content was measured using Spector’s method with bovine albumin as a standard [8,20]. To determine the best condition to polysaccharide acid hydrolysis using HCl, that is, with polysaccharide degradation without destruction of monosaccharides released, xylan was hydrolyzed with 0.5, 1, 2, and 4 M HCl, respectively, for various lengths of time (0.5, 1, 2 and 4 h), at 100 °C. The amount of released monosaccharides was then measured as described earlier [42] and the best hydrolysis condition determined (2 M HCl, 2 h). After acid hydrolysis, sugar composition was determined by the VWR-Hitachi LaChrom Elite^®^ HPLC system with an L-2490 refractive index detector. A LichroCART^®^ 250-4 column (250 mm × 40 mm) packed with Lichrospher^®^ 100 NH_2_ (5 μm) was coupled to the system. The sample mass used was 0.2 mg and analysis time was 25 min. The following sugars were analyzed as references: arabinose, galactose, glucose, glucuronic acid, fructose, fucose, mannose, *N*-acetyl-glucosamine, galactosamine, galactosamine, rhamnose, and xylose. Total phenolic compounds in xylan solution were determined using Folin-Ciocalteu’s colometric method and gallic acid as a standard [8]. Uronic acid content was determined by Dische [43]. The sulfate content was quantified after acid hydrolysis with HCl 4N for 6 h, 100 °C, then measured by gelatin-barium method previously described [40,41]. The xylan was carboxyl reduce using NaBH_4_ and 1-cyclohexyl-3-(2-morpholinoethyl)carbodiimide metho-*p*-toluenesulfonate as previously described [20].

### 3.3. Molecular Weight Determination

The xylan was subjected to gel-permeation chromatography on Sephadex G-100 (140 cm × 1 cm) using 0.2 M acetic acid as eluent. The elution was monitored for total sugar as described early [40]. To estimate the molecular weight of the polysaccharides, dextrans of different molecular weights were used as standards.

### 3.4. Fourier Transformed Infrared (FT-IR) Spectroscopy Analysis

Xylan (5 mg) was mixed thoroughly with dry potassium bromide. A pellet was prepared and the infrared spectra between 500 and 4000 cm^−1^ was measured on a Thermo-Nicolet Nexus 470 ESP FT-IR spectrometer. Thirty-two scans at a resolution of 4 cm^−1^ were averaged and referenced against air.

### 3.5. Antioxidant Activities

The antioxidant activity was examined by conducting of five tests *in vitro*: total antioxidant capacity, hydroxyl radical scavenging, superoxide radical scavenging, and ferric chelating, and reducing power as previously described [8].

#### 3.5.1. Determination of Total Antioxidant Capacity

The assay for total antioxidant capacity is based on the reduction of Mo^+6^ to Mo^+5^ by the polysaccharide and subsequent formation of a green phosphate/Mo^+5^ complex at acidic pH. The tubes containing the polysaccharides and reagent solution (0.6 M sulfuric acid, 28 mM sodium phosphate and 4 mM ammonium molybdate) were incubated at 95 °C for 90 min. After the mixture had cooled to room temperature, the absorbance of each solution was measured at 695 nm against a blank. The antioxidant capacity was expressed as mg of ascorbic acid/g of polysaccharide, described as an ascorbic acid equivalent.

#### 3.5.2. Hydroxyl Radical Scavenging Activity Assay

The hydroxyl radical scavenging activity of polysaccharides (from 0.1 to 2.0 mg/mL) was investigated using Fenton’s reaction (Fe^2+^ + H_2_O_2_ → Fe^3+^ + OH^−^ + OH^·^). These results were expressed as the rate of inhibition. Hydroxyl radicals were generated using a method described previously in 3 mL of 150 mM sodium phosphate buffer (pH 7.4) containing 10 mM FeSO_4_ × 7H_2_O, 10 mM EDTA, 2 mM sodium salicylate, 30% H_2_O_2_ and varying polysaccharide concentrations. In the control, sodium phosphate buffer replaced the H_2_O_2_. The solutions were incubated at 37 °C for 1 h, and the presence of the hydroxyl radical was detected by monitoring absorbance at 510 nm.

#### 3.5.3. Superoxide Radical Scavenging Activity Assay

The superoxide radical scavenging assay was based on the capacity of polysaccharides (from 0.1 to 2.0 mg/mL) to inhibit the photochemical reduction of nitroblue tetrazolium (NBT) in the riboflavin-light-NBT system. Each 3 mL reaction mixture contained 50 mM phosphate buffer (pH 7.8), 13 mM methionine, 2 mM riboflavin, 100 mM EDTA, 75 mM NBT, and 1 mL sample solution. The production of blue formazan was monitored as absorbance increased at 560 nm after a 10 min illumination with a fluorescent lamp. The entire reaction assembly was enclosed in a box lined with aluminum foil. Identical tubes with the reaction mixture were kept in the dark and served as the blank.

#### 3.5.4. Ferric Chelating

The ferrous ion chelating ability of samples (from 0.1 to 2.0 mg/mL) was investigated according to previously described. Briefly, the reaction mixture that contained samples of FeCl_2_ (0.05 mL, 2 mM) and ferrozine (0.2 mL, 5 mM) was mixed well and incubated for 10 min at room temperature. The absorbance of the mixture was measured at 562 nm against a blank.

#### 3.5.5. Reducing Power

Briefly, 4 mL of reaction mixture, containing different sample concentrations (from 0.1 to 2.0 mg/mL) in 0.2 M phosphate buffer (pH 6.6), was incubated with potassium ferricyanide (1% w/v) at 50 °C for 20 min. The reaction was stopped by addition of TCA solution (10% w/v). The solution was then mixed with distilled water and ferric chloride (0.1% w/v) solution, and the absorbance was measured at 700 nm. The results are expressed as a percentage of the activity shown by 0.2 mg/mL vitamin C.

### 3.6. Antiproliferative Activity

HeLa and 3T3 cells were grown in 75 cm^2^ flasks in DMEM medium. Cells were seeded into 96-well plates at a density of 5 × 10^3^ cell/well and allowed to attach overnight in 200 μL of medium at 37 °C and 5% CO_2_. The xylan was added at final concentrations of 0.5, 1.0, 1.5, and 2.0 mg/mL for 72 h at 37 °C and 5% CO_2_. After incubation, traces of polysaccharides were removed by washing the cells twice with 200 μL of PBS and 100 μL of fresh medium and 10 μL of 12 mM MTT (3-(4,5-Dimethylthiazol-2-yl)-2,5-diphenyltetrazolium bromide) dissolved in PBS was added to determine the effects of the polysaccharides on cell proliferation. The cells were then incubated for 4 h at 37 °C and 5% CO_2_. To solubilize the product of MTT reduced, 100 μL of isopropanol containing 0.04 N HCl was added to each well and thoroughly mixed using a multichannel pipettor. Within 1 h of HCl-isopropanol addition, the absorbance at 570 nm was read using a Multiskan Ascent Microplate Reader (Thermo Labsystems, Franklin, MA, USA). The percent of cell proliferation was calculated as follows:

(1) $$\%\;\text{cell proliferation}=\frac{\text{Abs}.570\;\text{nm of sample}}{\text{Abs}.570\;\text{nm of control}}\times 100$$

### 3.7. Anticoagulant Activity

Activated partial thromboplastin time (aPTT) and prothrombin time (PT) assay were performed according to manufacturers (Labtest, Minas-Gerais, Brazil). Briefly, citrated plasma was obtained by centrifugation of blood from adult, healthy and both sexes. It was verified by these tests, the mass of polysaccharide (100; 200; 400 and 600 μg) necessary to extend at least two times the normal clotting. Clexane, sulfated polysaccharide anticoagulant used commercially was used to comparison of the anticoagulant activity. The clotting time was determined using an automatic coagulometric Quick Time II, DRAKE, São Paulo, Brazil.

### 3.8. Antimicrobial Activity

#### 3.8.1. Antibiofilm Assay

The protocol was adapted from Antunes *et al.* [44], employing 96 well microtiter plates and crystal violet. In brief, 80 μL of the bacterial suspension (*Staphylococcus epidermidis*-ATCC 35984 and a clinical isolate of KPC producing *Klebsiella pneumonia* strain 174), 80 μL of the aqueous extract (0.5, 1.0, 1.5, and 2.0 mg/mL) and 40 μL of tryptone soya broth (TSB) (Oxoid Ltd., England) were added. Further, the plate was incubated (37 °C for 24 h) and the content of the wells removed and washed three times with sterile saline. The remaining attached bacteria were heat fixed at 60 °C for 1 h. The adherent biofilm formed was stained with 0.4% crystal violet for 15 min at room temperature. The crystal violet bound to the cells/biofilm was solubilized with 99.5% DMSO (Sigma-Aldrich Co., St. Louis, MO, USA) and absorbance was measured at 570 nm (Spectramax M2e Multimode Microplate Reader, Molecular Devices, Sunnyvale, CA, USA). The controls were considered 100% of biofilm formation and the values obtained for the extracts were a mean of triplicate experiments.

These two bacterial specie were chosen due to their clinical importance. The *S. epidermidis* is the main pathogen associated to catheter-infections [45] and KPC producing *Klebsiella pneumoniae* is a worldwide threat [46], since there are no effective antibiotic therapies against this pathogen.

#### 3.8.2. Bacterial Inhibition Assay

The experiment was performed as Trentin *et al.* [47]. The planktonic bacterial growth of *S. epidermidis* (ATCC 35984) and a clinical isolate of KPC producing *K. pneumoniae* was evaluated by the absorbance difference measured at OD600 at the end and at the beginning of the incubation time in polystyrene 96 well microtiter plates. Different concentrations of aqueous extract (0.5, 1.0, 1.5, and 2.0 mg/mL) were used in the presence of each bacterial strain. The control with water (instead extract) was considered to represent 100% of planktonic bacterial growth. All experiments were performed in triplicate.

### 3.9. Statistical Analysis

All data are expressed as means ± standard of quadruplicates measurements. Each experiment was performed at least three times. Statistical analysis was performed by one-way ANOVA using the SigmaStat [48], Student-Newmans-Keuls post-tests were performed for multiple group comparison. In all cases, statistical significance was set at *p* < 0.05.

## 4. Conclusion

Using a methodology that combines alkaline hydrolysis and ultrasound, we obtained a heteroxylan of 130 kDa. This polymer exhibited total antioxidant and high iron-chelating capacity. Moreover, it showed antiproliferative activity against human HeLa tumor cells and no activity in 3T3 normal cells. Corn cob xylan showed specific antimicrobial and anticoagulant activity, this last function is possibly related to carboxylic groups, making this polysaccharide a compound with multifunctional drug potential. It was concluded that corn cobs are an important source of natural bioactive compounds. Further studies will identify and purify active fractions from the corn cob xylan we extracted. The aim of these studies will be to increase our understanding of the complete structure of polysaccharides, including monosaccharide composition and sequence, glycosidic linkage configuration, glycosidic linkage position, branch point position, and the structure-function relationship. All of this knowledge will increase the likelihood of elucidating the biological roles of polysaccharides and develop potential antitumor and/or antioxidant drugs, based on three-dimensional structures.

## Figures and Tables

**Table 1:** Chemical composition of xylan extracted from corncobs.

Compound	Polysaccharides (%)	Phenolic Compounds (%)	Protein (%)	Molar Ratio a (%)					

				Xyl	Ara	Glc	Gal	Man	Glu A
Xylan	70	<0.01	0.4	50.0	15.0	20.0	10.0	2.5	2.5

Xyl: Xylose; Ara: Arabinose; Glc: Glucose; Gal: Galactose; Man: Mannose; Glu A: Glucuronic acid;

a Analyzed by HPLC after acid hydrolysis at 100 °C for 2 h.

**Table 2:** Anticoagulant activity in APTT test of xylan from corn cob a.

APTT (s) b			

Clexane^®^^c^		Xylan	

Mass Utilized (μg)	Clotting Time (s)	Mass Utilized (mg)	Clotting Time (s)
0.1	35.2 ± 3.5	0.1	69.5 ± 5.2
0.4	195.6 ± 8.8	0.2	117.0 ± 3.3
1.0	≥240	0.4	148.5 ± 2.4
10.0	≥240	0.6	214.8 ± 4.0

a The data are the mean values of tree determinations;

b APTT: activated partial thromboplastine time. For control sample without compound 35.1 ± 0.1 s;

c Clexane^®^: low molecular weight heparin.

**Table 3:** Antimicrobial activity of xylan from corn cob a.

Microorganism	Xylan From Corn Cob	

	% Antibacterial b,c	% Antibiofilm b,c
KPC producing *Klebsiella pneumoniae* (clinical isolate)	25.4 ± 0.02	30.0 ± 0.09
*Staphylococcus epidermidis* (ATCC 35984)	0	0

a The data are the mean values of three determinations ± SD;

b The results comes from the concentration of 2 mg/mL, other concentrations tested had no significant activity;

c Rifampicin (Sigma-Aldrich Co., St. Louis, MO, USA) was used as a control antibiotic.

## References

[1] Winterbourn C.C. (2008). Reconciling the chemistry and biology of reactive oxygen species. Nat. Chem. Biol.

[2] Koppula S.B., Ammani K. (2011). Antioxidant findings of araku environment medicinal plants using different assays. Drug Invent. Today.

[3] Soory M. (2009). Relevance of nutritional antioxidants in metabolic syndrome, ageing and cancer: Potential for therapeutic targeting. Infect. Disord. Drug Targets.

[4] Balsano C., Alisi A. (2009). Antioxidant effects of natural bioactive compounds. Curr. Pharm. Des.

[5] Sun J., Chu Y.F., Wu X., Liu R.H. (2002). Antioxidant and antiproliferative activities of common fruits. J. Agric. Food Chem.

[6] Camara R.B., Costa L.S., Fidelis G.P., Nobre L.T., Dantas-Santos N., Cordeiro S.L., Costa M.S., Alves L.G., Rocha H.A. (2011). Heterofucans from the brown seaweed *Canistrocarpus cervicornis* with anticoagulant and antioxidant activities. Mar. Drugs.

[7] de Sousa A.P.A., Torres M.R., Pessoa C., de Moraes M.O., Filho F.D.R., Alves A.P.N.N. (2007). *In vivo* growth-inhibition of Sarcoma 180 tumor by alginates from brown seaweed *Sargassum vulgare*. Carbohydr. Polym.

[8] Costa L.S., Fidelis G.P., Cordeiro S.L., Oliveira R.M., Sabry D.A., Camara R.B., Nobre L.T., Costa M.S., Almeida-Lima J., Farias E.H. (2010). Biological activities of sulfated polysaccharides from tropical seaweeds. Biomed. Pharmacother.

[9] da Silva T.D., Giordani R.B., Zimmer K.R., da Silva A.G., da Silva M.V., Correia M.T., Baumvol I.J.R., Macedo A.J. (2011). Potential of medicinal plants from the Brazilian semi-arid region (Caatinga) against Staphylococcus epidermidis planktonic and biofilm lifestyles. J. Ethnopharmacol.

[10] Mehrotra V., Mehrotra S., Kirar V., Shyam R., Misra K., Srivastava A.K., Nandi S.P. (2011). Antioxidant and antimicrobial activities of aqueous extract of Withania somnifera against methicillin-resistant *Staphylococcus aureus*. J. Microbiol. Biotechnol. Res.

[11] Ebringerová A., Kardosová A., Hromádková Z., Malovíková A., Hríbalova V. (2002). Immunomodulatory activity of acidic xylans in relation to their structural and molecular properties. Int. J. Biol. Macromol.

[12] Anwar F., Jami A., Iqbal S., Sheikh M.A. (2006). Antioxidant activity of various plant extracts under ambient and accelerated storage of sunflower oil. Grasas y Aceites.

[13] Garcia R.B., Ganterb J.L.M.S., Carvalho R.R. (2000). Solution properties of d-xylans from corn cobs. Eur. Polym. J.

[14] Wang Y., Zhang J. (2006). A novel hybrid process, enhanced by ultrasonication, for xylan extraction from corncobs and hydrolysis of xylan to xylose by xylanase. J. Food Eng.

[15] Hromadková Z., Kovaciková J., Ebringerová A. (1999). Study of the classical and ultrasound-assisted extraction of the corn cob xylan. Ind. Crops Prod.

[16] Ebringerová A., Hromadková Z., Alfoldi J., Berth G. (1992). Structural and solution properties of corn cob heteroxylans. Carbohydr. Polym.

[17] Ebringerova A., Hromadkova Z., Alfoldi J., Hribalova V. (1998). The immunologically active xylan from ultrasound-treated corn cobs: Extractability, structure and properties. Carbohydr. Polym.

[18] Ebringerová A., Hromádková Z. (1997). The effect of ultrasound on the structure and properties of the watersoluble corn hull heteroxylan. Ultrason. Sonochem.

[19] Garcia R.B., Nagashima T., Praxedes A.K.C., Raffin F.N., Moura T.F.A.L., do Egito E.S.T. (2001). Preparation of micro and nanoparticles from corn cobs xylan. Polym. Bull..

[20] Barroso E.M.A., Costa L.S., Medeiros V.P., Cordeiro L.S., Costa M.S.S.P., Franco C.R.C., Nader H.B., Leite E.L., Rocha H.A.O. (2008). A non-anticoagulant heterofucan has antithrombotic activity *in vivo*. Planta Med.

[21] Kacuráková M., Wellner N., Ebringerová A., Hromádková Z., Wilson R.H., Belton P.S. (1999). Characterisation of xylan-type polysaccharides and associated cell wall components by FT-IR and FT-Raman spectroscopies. Food Hydrocoll.

[22] Oliveira E.E., Silva A.E., Nagashima T., Gomes M.C.S., Aguiar L.M., Marcelino H.R., Araujo I.B., Bayer M.P., Ricardo N.M.P.S., Oliveira A.G. (2010). Xylan from corn cobs, a promising polymer for drug delivery production and characterization. Bioresour. Technol.

[23] Prieto P., Pineda M., Aguilar M. (1999). Spectrophotometric quantitation of antioxidant capacity through the formation of a phosphomolybdenum complex: Specific application to the determination of vitamin E. Anal. Biochem.

[24] Choi J.-I., Kim J.-K., Srinivasan P., Kim J.-H., Park H.-J., Byun M.-W., Lee J.-W. (2009). Comparison of gamma ray and electron beam irradiation on extraction yield, morphological and antioxidant properties of polysaccharides from tamarind seed. Radiat. Phys. Chem.

[25] Singh S., Singh R.P. (2008). *In Vitro* methods of assay of antioxidants: An overview. Food Rev. Int.

[26] Cao Y., Ikeda I. (2009). Antioxidant activity and antitumor activity (*in vitro*) of xyloglucan selenious ester and surfated xyloglucan. Int. J. Biol. Macromol.

[27] Wang J., Zhang Q., Zhang Z., Li Z. (2008). Antioxidant activity of sulfated polysaccharide fractions extracted from *Laminaria japonica*. Int. J. Biol. Macromol.

[28] Qi H., Zhao T., Zhang Q., Li Z., Zhao Z., Xing R. (2005). Antioxidant activity of different molecular weight sulfated polysaccharides from *Ulva pertusa* Kjellm (Chlorophyta). J. Appl. Phycol.

[29] Zhou Y.C., Zheng R.L. (1991). Phenolic compounds and an analog as superoxide anion scavengers and anti oxidants. Biochem. Pharmacol.

[30] Liu W., Wang H., Yao W., Gao X., Yu L. (2010). Effects of sulfation on the physicochemical and functional properties of a water insoluble polysaccharide preparation from*Ganoderma lucidum*. J. Agric. Food Chem.

[31] Changa S.C., Hsua B.Y., Chen B.H. (2010). Structural characterization of polysaccharides from *Zizyphus jujube* and evaluation of antioxidant activity. Int. J. Biol. Macromol.

[32] Telles C.B.S., Sabry D.A., Almeida-Lima J., Costa M.S.S.P., Melo-Silveira R.F., Trindade E.S., Sassaki G.L., Wisbeck E., Furlan S.A., Leite E.L. (2011). Sulfation of the extracellular polysaccharide produced by the edible mushroom *Pleurotus sajor* caju alters its antioxidant, anticoagulant and antiproliferative properties *in vitro*. Carbohydr. Pol.

[33] Yang X.B., Gao X.D., Han F., Tan R.X. (2005). Sulfation of a polysaccharide produced by marine filamentous fungus *Phoma herbarum* YS4108 alters its antioxidant properties *in vitro*. Biochim. Biophys. Acta.

[34] Ryu D.S., Kim S.H., Lee D.S. (2009). Anti-proliferative effect of polysaccharides from *Salicornia herbacea* on induction of G2/M arrest and apoptosis in human colon cancer cells. J. Microbiol. Biotechnol.

[35] Ando H., Ohba H., Sakak I.T., Takamine K., Kamino Y., Moriwaki S., Bakalova R., Uemura Y., Hatate Y. (2004). Hot-compressed-water decomposed products from bamboo manifest a selective cytotoxicity against acute lymphoblastic leukemia cells. Toxicol. in Vitro.

[36] Meyers K.J., Watkins C.B., Pritts M.P., Rui H.L. (2003). Antioxidant and antiproliferative activities of strawberries. J. Agric. Food Chem.

[37] Yoon S., Pereira M.S., Pavao M.S.G., Hwang J., Pyun Y., Mourao P.A.S. (2002). The medicinal plant *Porana volubilis* contains polysaccharides with anticoagulant activity mediated by heparin cofactor II. Thromb. Res.

[38] Lee C.K., Kin H., Moon K.H., Shun K.H. (1998). Screening and isolation of antibiotic resistance inhibitors from herb materials resistance inhibition of volatile components of Korean aromatic herbs. Arch. Pharm. Res.

[39] Gul M.Z., Bhakshu L.M., Ahmad F., Kondapi A.K., Qureshi I.A., Ghazi I.A. (2011). Evaluation of *Abelmoschus moschatus* extracts for antioxidant, free radical scavenging, antimicrobial and antiproliferative activities using *in vitro* assays. BMC Complement. Altern. Med.

[40] Almeida-Lima J., Costa L.S., Silva N.B., Melo-Silveira R.F., Silva F.V., Felipe M.B., Medeiros S.R., Leite E.L., Rocha H.A. (2010). Evaluating the possible genotoxic, mutagenic and tumor cell proliferation-inhibition effects of a non-anticoagulant, but antithrombotic algal heterofucan. J. Appl. Toxicol.

[41] Rocha H.A., Bezerra L.C., de Albuquerque I.R., Costa L.S., Guerra C.M., de Abreu L.D., Nader H.B., Leite E.L. (2005). A xylogalactofucan from the brown seaweed *Spatoglossum schröederi* stimulates the synthesis of an antithrombotic heparan sulfate from endothelial cells. Planta Med.

[42] Magalhaes K.D., Costa L.S., Fidelis G.P., Oliveira R.M., Nobre L.T.D.B., Dantas-Santos N., Camara R.B.G., Albuquerque I.R.L., Cordeiro S.L., Sabry D.A. (2011). Anticoagulant, antioxidant and antitumor activities of heterofucans from the seaweed *Dictyopeteris delicatula*. Int. J. Mol. Sci.

[43] Dische Z. (1974). A new specific color reactions of hexuronic acids. J. Biol. Chem.

[44] Antunes A.L., Trentin D.S., Bonfanti J.W., Pinto C.C., Perez L.R., Macedo A.J., Barth A.L. (2010). Application of a feasible method for determination of biofilm antimicrobial susceptibility in staphylococci. APMIS.

[45] Antunes A.L., Bonfanti J.W., Perez L.R., Pinto C.C., Freitas A.L., Macedo A.J., Barth A.L. (2011). High vancomycin resistance among biofilms produced by *Staphylococcus* species isolated from central venous catheters. Mem. Inst. Oswaldo Cruz.

[46] Gupta N., Limbago B.M., Patel J.B., Kallen A.J. (2011). Carbapenem-resistant enterobacteriaceae: Epidemiology and prevention. Clin. Infect. Dis.

[47] Baumvol I.J., Macedo A.J. (2011). Potential of medicinal plants from the Brazilian semi-arid region (Caatinga) against *Staphylococcus epidermidis* planktonic and biofilm lifestyles. J. Ethnopharmacol.

[48] (1997). *SigmaStat*, version 2.01.

